# Highly Flexible Poly(1,12-dodecylene 5,5′-isopropylidene-bis(ethyl 2-furoate)): A Promising Biobased Polyester Derived from a Renewable Cost-Effective Bisfuranic Precursor and a Long-Chain Aliphatic Spacer

**DOI:** 10.3390/molecules28104124

**Published:** 2023-05-16

**Authors:** Sami Zaidi, Abdelkader Bougarech, Majdi Abid, Souhir Abid, Armando J. D. Silvestre, Andreia F. Sousa

**Affiliations:** 1 CICECO–Aveiro Institute of Materials and Department of Chemistry, University of Aveiro, 3810-193 Aveiro, Portugal; 2 Faculty des Sciences, Laboratory de Chimie Appliquée HCGP, Université de Sfax, Sfax 3038, Tunisia; 3 Chemistry Department, College of Science and Arts in Al-Qurayyat, Jouf University, Al-Qurayyat P.O. Box 756, Al Jouf, Saudi Arabia; 4 CEMMPRE, Department of Chemical Engineering, University of Coimbra, 3030-790 Coimbra, Portugal

**Keywords:** bisfuranic building-block monomers, 1,12-dodecandiol, biobased polyesters, flexible materials

## Abstract

The continuous search for novel biobased polymers with high-performance properties has highlighted the role of monofuranic-based polyesters as some of the most promising for future plastic industry but has neglected the huge potential for the polymers’ innovation, relatively low cost, and synthesis easiness of 5,5′-isopropylidene bis-(ethyl 2-furoate) (DEbF), obtained from the platform chemical, worldwide-produced furfural. In this vein, poly(1,12-dodecylene 5,5′-isopropylidene -bis(ethyl 2-furoate)) (PDDbF) was introduced, for the first time, as a biobased bisfuranic long-chain aliphatic polyester with an extreme flexibility function, competing with fossil-based polyethylene. This new polyester in-depth characterization confirmed its expected structure (FTIR, ^1^H, and ^13^C NMR) and relevant thermal features (DSC, TGA, and DMTA), notably, an essentially amorphous character with a glass transition temperature of −6 °C and main maximum decomposition temperature of 340 °C. Furthermore, PDDbF displayed an elongation at break as high as 732%, around five times higher than that of the 2,5-furandicarboxylic acid counterpart, stressing the unique features of the bisfuranic class of polymers compared to monofuranic ones. The enhanced ductility combined with the relevant thermal properties makes PDDbF a highly promising material for flexible packaging.

## 1. Introduction

In the past decades, an increasing social, environmental, legislative, and ultimately economic demand for a more sustainable model of development [1] has witnessed a growing interest in renewable-based polymers to replace their analogous from fossil origin, namely the highly consumed polyethylene (PE), polypropylene (PP), poly(ethylene terephthalate) (PET), and polystyrene (PS), among other thermoplastics [2,3,4,5,6].

In this vein, ubiquitous biomass fractions such as cellulose, lignin, polysaccharides, proteins, and vegetable oils have emerged as an inexhaustible source of raw materials to prepare a wide portfolio of valuable, and, in many cases, innovative building-block chemicals for polymer synthesis. Among them, 2,5-furandicarboxylic acid (FDCA) and various alkyl 2-furoates (including bisfuranic derivatives) are some of the most important [7,8,9,10,11]. While FDCA has been extensively used [11,12], mainly due to its structural similarity to fossil-based terephthalic acid and a set of unique feature it can impart to polymers thereof (e.g., enhanced barrier properties) [13,14,15], opposite, bisfuran monomers have been widely overlooked.

FDCA has been exploited in both fully aromatic polymers (mainly polyesters) [16,17,18,19] as well as in aliphatic-furanic ones [20,21,22,23,24]. The most popular class is undoubtedly the linear-aliphatic polyesters [12], with poly(ethylene 2,5-furandicarboxylate) (PEF) and poly(1,3-propylene 2,5-furandicarboxylate) (PPF) occupying a preeminent place as biobased alternatives to the petrochemical PET in films, fibers, and packaging [5]. Additionally, other poly(alkylene 2,5-furandicarboxylate)s with longer alkyl chain-length have also been extensively investigated to modulate the flexibility, crystallization kinetics, and (bio)degradability, and, hence, to expand their overall use in, for example, packaging [25,26].

Of particular interest are those with very long alkyl-chains (≥C8) due to the combination of rigidity imparted by the furan ring and the extreme softness associated with the aliphatic moieties. Despite this feature, there are only a few reports on this type of polyesters, focusing mostly on the optimization of their synthesis or in the study of their thermal properties [25,27,28,29,30,31]. The high boiling points of the very long-chain diols is one of the serious problems that hinders their synthesis by the classical polycondensation procedures via the evaporation of the excess of diols [30]. To circumvent this issue, in 2015, the Bikiaris group, developed an elegant approach to synthesize high-molecular-weight polyesters from these type of monomers, simply by reacting dimethyl 2,5-furandicarboxylate (DMFDC) with the long-chain diols to form the correspondent bis(hydroxyalkyl)-2,5-furandicarboxylates, followed by the addition of a slight excess of DMFDC (1.05 eqv.) [27]. Later, in 2017, Li et al. [25] proposed a different strategy based on the use of asymmetric 2-(hydroxyalkyl)-5-methyl 2,5-furandicarboxylate monomer, prepared from FDCA, but following a highly unsustainable approach using acyl chloride intermediate.

Other works focused instead on the assessment of properties of such polyesters [25,26,28]; for example, Feherenbacher et al. [32] early reported, in 2009, on the thermal characterization of poly(1,6-hexylene 2,5-furandicarboxylate) (PHF), poly(1,12-dodecylene 2,5-furandicarboxylate) (PDDF), and poly(1,18-octadecylene 2,5-furandicarboxylate) (PODF). Importantly, they found that these polyesters are semi-crystalline polymers with a melting feature decreasing with the increasing chain length. These results were later corroborated by the work of Bikiaris and Papageorgiou [27]. Notably, PDDF displayed a melting temperature (T_m_) of 111 °C and a sub-ambient glass transition temperature (T_g_~−22 °C). Despite the relatively moderate melting and glass transition temperatures, FDCA-based long-chain aliphatic polyesters display a combination of (bio)degradation and mechanical properties, which makes them suitable for a broad range of applications [24,26]. More recently, nanocomposite films prepared by blending poly(lactic acid) (PLA), PDDF (10 wt%), and reduced graphene oxide as filler were studied as new promising biobased materials for packaging [31]. Fibers prepared from PLA/PDDF blends for textile applications have also been reported [33].

Less extensive research has been dedicated to bisfurans, such as 5,5′-isopropylidene bis-(ethyl 2-furoate) (DEbF), obtained with high purity, through a simple and cost-effective process from the platform chemical furfural [34,35], albeit their huge potential for the polymers’ innovation, relatively low cost [36], and synthesis easiness compared to FDCA [37]. DEbF has been readily prepared in optimized conditions, simply from the condensation of biobased ethyl 2-furoate with acetone in acidic medium [38]. Entirely aromatic polyesters bearing bisfuranic and pyridinic units in the main chain display high T_g_ and amorphous character [39], opposite to FDCA-based counterparts, which are typically semi-crystalline materials. Ionic polyesters containing sulfonated functions and bisfuranic moieties have sorption and hydrolytic degradation features [40,41]. High molecular weight aliphatic-bisfuranic polyesters, obtained through the interfacial polycondensation of the bisfuranic dichloride derivative 5,5′-isopropylidene bis-(2-furoyl chloride), with C2 to C6 diols (ethylene glycol, 1,4-butanediol and 1,6-hexandiol), typically exhibited good stability and moderate T_g_, showing the same typical trend already mentioned for FDCA-based polymers decreasing with the increasing number of methylene groups [6]. To the best of our knowledge, the mechanical properties of this type of polymer have not been reported before, despite the importance of this functional property for the assessment of potential applications.

Additionally, to the best of our knowledge, bisfuranic long-chain aliphatic polyesters have never been reported before, albeit their anticipated enhanced flexibility, imparted by the combination of a long-aliphatic chain spacer and the less-rigid DEbF bisfuranic structure, with a symmetrical carbon between the two furan rings. This kind of polyester will be of interest for flexible packaging, an ever-growing sector [42]. Therefore, in the current study, the unclosed potential of biobased long-chain aliphatic-bisfuranic polyesters was exploited for the first time, by synthesizing the novel poly(1,12-dodecylene 5,5′-isopropylidene-bis(ethyl 2-furoate)) (PDDbF), using a straightforward polycondensation approach. PDDbF in-depth structural (FTIR and ^1^H and ^13^C NMR), thermal (TGA, DSC), and mechanical (tensile testing and DMTA) properties were assessed.

## 2. Results and Discussions

**Scheme 1:**
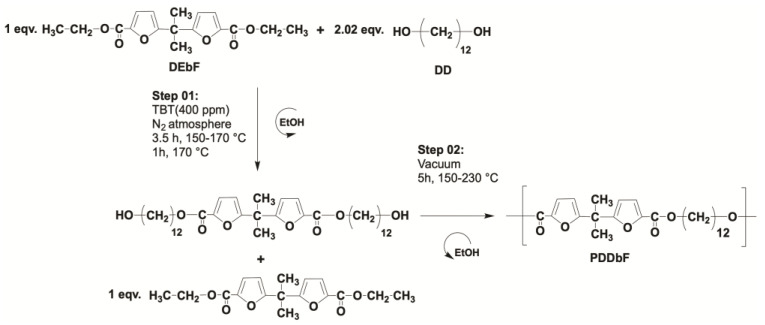
Synthesis of PDDbF.

In order to evaluate the unexploited influence of the chain length on bisfuranic polymers properties, this study addresses, for the first time, the synthesis and characterization of biobased poly(1,12-dodecylene 5,5′-isopropylidene-bis(ethyl 2-furoate)). PDDbF comprises both stiff units (5,5′-isopropylidene-bis(ethyl 2-furoate)) and soft aliphatic chain (1,12-dodecylene), promptly synthesized by applying a two-step melt polycondensation procedure, as described in the experimental section and depicted in Scheme 1. The PDDbF isolation yield after purification was over 75% and the high intrinsic viscosity ($\left[\eta \right]\approx 0.63$ dL.g^−1^) was found to be in accordance with a high molecular weight polymer ($M_{v}$ of 40,000). Highly supple films, with c.a. 700–800 μm thickness, prepared by polyester melting/cooling process, pave the way to assess, for the first-time, the bisfuranic-based materials’ mechanical properties.

### 2.1. Structural Characterization

**Figure 1:**
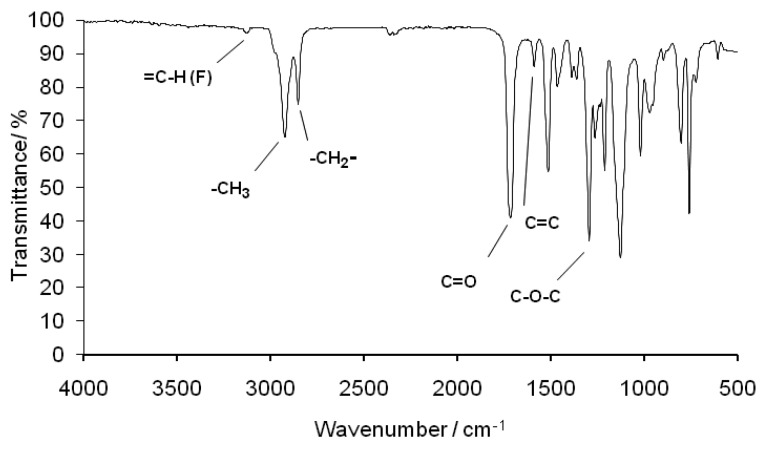
PDDbF FTIR spectrum.

**Figure 2:**
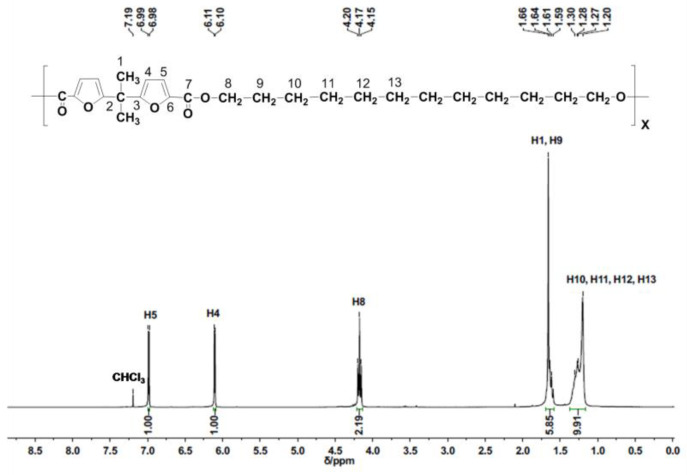
^1^H NMR spectrum of PDDbF.

The detailed chemical structure of PDDbF was studied using FTIR, ^1^H and ^13^C NMR spectroscopies (Figure 1 and Figure 2) and compared to the monomers’ counterparts (Figures S1–S3). The FTIR spectrum of Figure 1 displays all the typical characteristic bands for furan rings. Namely, a small band at 3128 cm^−1^ assigned to =C-H stretching vibration, an intense peak near 2922 cm^−1^ characteristic of the stretching mode of the methyl groups, a band at 1590 cm^−1^ related to the C=C stretching, a very intense peak near 1127 cm^−1^ arising from the C-O-C furan ring vibration, and a band at 1020 cm^−1^ assigned to the furan ring breathing and several bands (971, 803 and 759 cm^−1^) related to 2,5-disubstituted furans. In addition, the FTIR spectrum also exhibits a band at 2852 cm^−1^ associated to the C-H stretching of the methylene groups of the aliphatic diol moiety and two very intense bands near 1716 and 1295 cm^−1^ assigned to the C=O and C-O-C stretching vibrations of ester groups, respectively.

The structure of PDDbF was also studied by ^1^H NMR (Figure 2 and Table S1 of the Supplementary Materials). As expected for (hetero-)aromatic-based polyesters, the most deshielded protons of PDDbF are those from the furanic rings [39,43,44], namely two doublets at 6.98 and 6.10 ppm attributed to the protons of furan heterocycle (H5 and H4, respectively). The resonance assigned to the methylene protons (H8) directly attached to the carbonyl group appears at 4.15–4.20 ppm as a triplet. The other proton resonances of the aliphatic segments are displayed at: 1.59–1.64 ppm (H9, multiplet), partially overlapped with the singlet at 1.66 ppm (H1), and between 1.20–1.30 ppm (H10, H11, H12, H13). Notably, the ^1^H NMR spectrum shows the high purity of PDDbF and the equivalence in terms of integration. Additionally, the PDDbF ^13^C NMR spectrum (Figure S4 and Table S2 of the Supplementary Materials) corroborates the previous assessment, with the detection of the following typical carbon resonances: 163.1 ppm arising from the carbonyl carbon (C7); several resonances at 158.9, 134.7, 118.6, 107.3, 38.3, and 25.9 ppm assigned to the furan ring carbons (C6, C3, C5, C4, C2 and C1, respectively); and at 64.9, 29.6, 29.5, 29.3, 28.7, and 26.0 ppm for the 1,12-dodecylene chain carbon resonances (C8, C9, C10, C11, C12, and C13).

### 2.2. Thermal and X-ray Diffraction Properties

**Figure 3:**
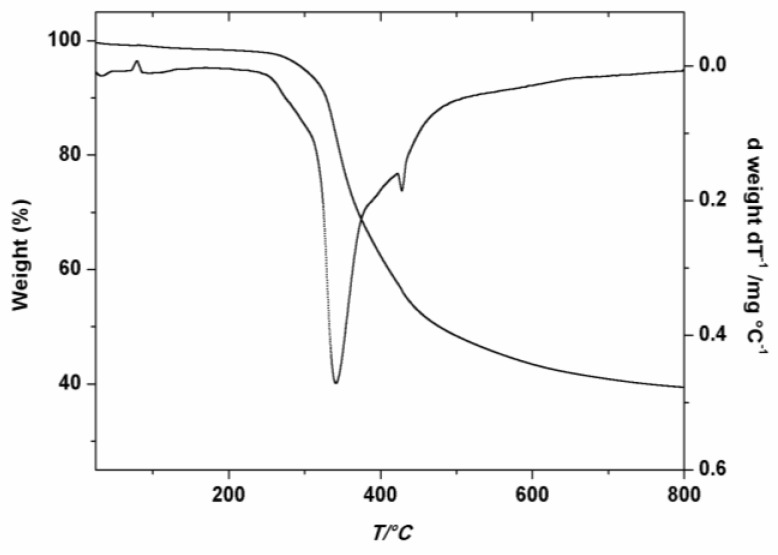
TGA and DTG thermograms of PDDbF.

The thermal stability of the as prepared PDDbF was evaluated by TGA. The thermogram of Figure 3 exhibits a major characteristic feature, at a maximum decomposition temperature (T_d,max_) of 341 °C (c.a. 83% weight loss), followed by a smaller peak near 430 °C. Comparing with linear low density poly(ethylene) (LLDPE), the thermal stability of PDDbF is much higher (T_d,max_, c.a. 341 vs. 250 °C) [44].

The thermal behavior of the PDDbF below the degradation temperature was studied by DSC analysis. The heating thermogram (Figure S5 of the Supplementary Materials) agrees with an essentially amorphous character, displaying accordingly, only one step in the baseline, ascribed to the glass transition temperature (T_g_) at −6 °C (also in agreement with DMTA results described below). This moderately low T_g_ is due to the high number of methylene groups in the aliphatic chain, which is associated to the high flexibility of the macromolecular chain. Nevertheless, this T_g_ is still higher than that of LLDPE [45]. This effect has never been exploited for the promising family of poly(5,5′-isopropylidene-bis(2-furoate))s, although long aliphatic chain FDCA derivatives, such as poly(1,12-dodecylene 2,5-furandicarboxylate) homologous (PDDF) [27] or poly(1,20-eicosanediyl 2,5-furandicarboxylate) (PM20F) [29], also have low T_g_s (Table 1). The DSC thermogram also shows a small melting peak at 81.6 °C. However, the enthalpy associated to such transition is much lower than that of PDDF with the same number of methylene groups (9 J g^−1^ vs. 50 J g^−1^), which is indicative of a lower crystallinity degree and that the crystallization of PDDbF is hampered by the bisfuranic moiety [27].

The XRD diffractogram (Figure S6 of the Supplementary Materials) of PDDbF presents only a halo centered at 2θ~19.2°. Polymers bearing the bisfuran motif follow a similar trend, being typically amorphous materials [6,39,41], opposite to the FDCA-based counterpart, poly(1,12-dodecylene 2,5-furandicarboxylate), which as mentioned above, is semi-crystalline [27]. The amorphous nature of bisfuranic polymers is most likely related to the myriad of different spatial arrangements of both furan rings hampering crystallization, opposite to FDCA-based ones.

### 2.3. DMTA and Mechanical Properties

**Figure 4:**
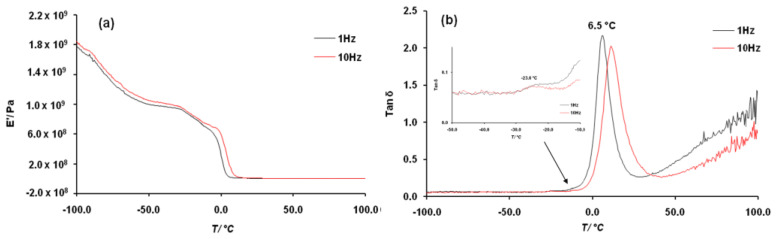
DMTA storage modulus (E′) (**a**) and tan δ (**b**) traces of PDDbF.

Dynamic mechanical behavior of PDDbF was investigated by DMTA and its storage modulus (E′) (Figure 4a), loss modulus (E″) (Figure S7 of the Supplementary Materials), and tan δ (Figure 4b) traces were recorded. At sub-ambient temperatures, c.a. −24 °C, the E’ trace shows a first small decrease of the modulus typically associated with an increase of chain degrees of freedom and most probably ascribed to the β relaxation, followed by a rapid drop attributed to an α relaxation, the T_g_ (c.a. 6 °C).

Importantly, the E’ value, measured at room temperature (20 °C) and in the region of the glass transition (4.07 × 10^6^ and 1.86 × 10^7^ Pa, respectively), allowed to study the elastic behavior of PDDbF. Typically, this polyester had a lower E’ than other aromatic polymers incorporating shorter aliphatic moieties (e.g., PET and PEF), hence being less stiff [48].

The tan δ trace (Figure 4b) obtained in the single cantilever mode agrees with the transitions mentioned above, displaying two peaks centered at approximately −24 and 6 °C attributed to T_β_ and T_g_, respectively. After the glass transition, the chain segments movement began to be easier, reaching a viscous liquid, which explain the loose increase of the tan δ value.

**Figure 5:**
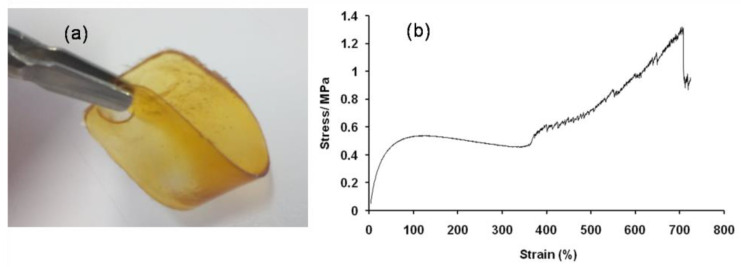
Digital image of a PDDbF film (**a**) and corresponding stress–strain curve (**b**).

Additionally, PDDbF compression molded films were subjected to stress–strain measurements (Figure 5) in order to explore their mechanical properties and assess their suitability to practical applications in, for example, flexible packaging [49]. Despite the evident importance of mechanical properties for the applications’ evaluation, to the best of our knowledge, this is the first study reporting these features for bisfuranic polyesters. 

The elongation at break, tensile stress at break, tensile stress at yield, and Young’s modulus of PDDbF, and of similar FDCA-based polyesters, are summarized in Table 2.

PDDbF exhibits a low Young’s modulus, above 2 MPa, but the elongation at break reached 732%, a value much higher than that of its FDCA counterpart (130%) with similar molecular weight [27], which is indicative of the role played by the bisfuranic moiety in the very flexible and easily deformable nature of the material obtained compared to the FDCA-based counterparts (PDDF and PDeF, Table 2) or even to PE [50]. Thus, considering also the PDDF values (Table 2), the explanation for the high elongation at break for PDDbF is not only due to the long-chain diol spacer but also due to the lower rigidity of the 5,5′-isopropylidene-bis(ethyl 2-furoate) moiety, most probably related to the symmetric carbon between the two furan rings, which results in a greater unfolding stretchability during deformation. Furthermore, the typical high stress at yield, high stress at break, and low elongation at break of furanoate-based polyesters compared to those of PDDbF can be explained by fact that the crystallites appear to serve a stress-bearing phase during deformation [51].

## 3. Materials and Methods

### 3.1. Materials

1,12-Dodecandiol (DD, 99%), ethyl 2-furoate (99%), titanium (IV) tert-butoxide (TBT, 97%), deuterated dimethyl sulfoxide (DMSO-d_6_, 99.5% D), and deuterated chloroform (CHCl_3_-d, 99.8% D) were purchased from Merck (Darmstadt, Germany). All solvents used were analytical grade. All chemicals were used as received, without further purification.

### 3.2. Preparation of 5,5′-Isopropylidene-bis(ethyl 2-furoate) (DEbF)

DEbF was synthesized according to a procedure described elsewhere [44]. Briefly, DEbF was prepared by reacting ethyl 2-furoate (0.214 mol) with acetone under acidic conditions, at 60 °C for 8 h. The monomer was then isolated as fine crystals after extraction with ethyl ether, followed by distillation and recrystallization from hexane. The structure and purity were confirmed by FTIR and ^1^H and ^13^C NMR spectroscopic techniques (Figures S1–S3 of the Supplementary Materials).

### 3.3. Synthesis of Poly(1,12-dodecylene 5,5′-isopropylidene-bis(ethyl 2-furoate)) (PDDbF)

PDDbF was synthesized by melt polycondensation, according to a previously reported procedure [29]. In the first step, stoichiometric amounts of DEbF (5 g, 15.6 mmol), an excess of DD (6.4 g, 31.5 mmol) and TBT (6.5 mg, 19.2 × 10^−3^ mmol) were charged into the glass reactor. The reaction started by heating progressively from 150 to 170 °C, during 3.5 h, and kept at that maximum temperature, under a nitrogen atmosphere, for 1 h. Before starting the second step, the reaction temperature was decreased to 150 °C, and an additional quantity of DEbF (5 g, 15.6 mmol) was added and the temperature was slowly increased again until 170 °C under vacuum for 3 h. Afterwards, the temperature was raised to 210 °C, and then, during 2 h, progressively increased up to 230 °C and kept at that maximum temperature for at least half hour. Finally, the obtained product was dissolved in chloroform, precipitated in an excess of cold methanol, filtered, and dried. PDDbF film specimens were prepared by melting procedure at c.a. 165 °C, placing the samples in a Teflon rectangular mold (50 × 10 × 1 mm^3^).

### 3.4. Characterization

Attenuated total reflection Fourier transform infrared (ATR FTIR) spectra were obtained using a PARAGON 1000 PerkinElmer FTIR spectrophotometer equipped with a single horizontal Golden Gate ATR cell. The resolution was 8 cm^−1^ after 128 scans in the range of 500–4000 cm^−1^.

^1^H and ^13^C Nuclear Magnetic Resonance spectroscopy (^1^H, ^13^C NMR) analyses of samples dissolved in CDCl_3_ were recorded using a Bruker AMX 300 spectrometer operating at 300 or 75 MHz. All chemical shifts were expressed in parts per million (ppm) and reported downfield from 0.00 ppm using tetramethylsilane (TMS) as an internal reference.

Intrinsic viscosity $\left[\eta \right]$ measurements were performed using an Ubbelohde-type viscometer, in a water bath at 25 °C. A mixture of phenol/1,1,2,2-tetrachloroethane (50/50) (wt%/wt%) was used as solvent and at a polymer concentration of 0.1 g per 20 mL of solution. The intrinsic viscosity was determined by the ratio of specific viscosity and sample solution concentration: $\eta_{sp}/c$, where $\eta_{sp}=(t_{1}-t_{0})/t_{0}$ and $t_{0}$ and $t_{1}$ are the solvent mixture and polyester solution elution time, respectively. The viscosity-average molecular weight ($M_{v}$) of PDDbF was calculated by the Mark–Houwink equation using the K and α parameters for PET viscosity $\left[\eta \right]=4.68\times 10^{-4}M_{v}^{0.68}$ [52,53,54].

Thermogravimetric analyses (TGA) were carried out with a Shimadzu TGA50 analyzer equipped with a platinum cell using platinum pans to encapsulate the samples (ca. 7 mg). Thermograms were recorded under a nitrogen flow of 20 mL min^−1^ at a constant heating rate of 10 °C min^−1^ from 25 to 800 °C.

DSC thermograms were obtained with a Setaram DSC92 calorimeter. Scans were carried out under dry nitrogen, with a heating rate of 10 °C min^−1^ in the temperature range of −50 to 250 °C. Two heating/cooling cycles were performed. The first heating scan was used to erase the thermal history of the polymer, and the second heating scan was performed after rapid cooling from the melted polymer.

X-ray diffraction (XRD) patterns were acquired using a Philips X’pert MPD diffractometer operating with CuKα radiation (λ = 1.5405980 Å) at 45 kV and 40 mA. Samples were scanned in the 2θ range of 3 to 60 °C, with a step size of 0.026° and a time per step of 69 s.

Dynamical mechanical thermal analysis (DMTA) of thick samples (5.0 × 3.6 × 0.8 mm^3^) were recorded with a Tritec 2000 DMTA Triton equipment operating in the single cantilever bending geometry. Tests were performed in multifrequency mode (1 and 10 Hz), from −100 to 200 °C, at 2 °C.min^−1^.

Tensile mechanical tests were performed on an Instron 5966 Series machine. A load cell of 500 N was used and operated at a deformation rate of 10 mm.min^−1^, under ambient conditions. For each sample, at least 5 specimens were tested. Young’s modulus, tensile strength, and elongation at break were calculated using the Bluehill 3 material testing software.

## 4. Conclusions

This study highlights the possibility of preparing a new biobased homopolyester from the bisfuranic monomer DEbF and a long-chain aliphatic diol spacer adopting a very straightforward approach based on the two-step polymerization approach. However, most importantly, this study uncloses these monomers’ ability to impart enhanced flexibility to the ensuing polymer chain compared to FDCA counterparts, but still having higher thermal stability and glass transition compared to polyolefins. In fact, the in-depth characterization of PDDbF showed that this polyester is amorphous, with a sub-ambient glass transition temperature (around −6 °C), and a maximum decomposition temperature of 341.4 °C, much higher than the same parameter for LLDPE (341 vs. 250 °C, respectively) [44]. All in all, PDDbF is a promising biobased polyester from the bisfuranic family and shows an enhanced elongation at break value of about 732%, thus opening their wider exploration among, for example, flexible packaging, an ever-growing sector.

## Figures and Tables

**Table 1:** Thermal properties of the synthesized PDDbF compared to PDDF, PM20F, and LLDPE.

Polyester	T_d,max_/°C ^a^	T_g_/°C ^b^	T_m_/°C ^c^	References
PDDbF	341, 430	−6	81.8	this work
PDDF	-	−5 ^c^	111	[46]
PM20F	407	8	107	[29]
LLDPE	250	−15	122	[44,45,47]

^a^ T_d,max_: temperature at which the rate of weight loss is maximum (DTG). ^b^ T_g_: glass transition temperature. ^c^ T_m_: melting temperature determined by DSC at 10 °C.min^−1^.

**Table 2:** Mechanical properties of PDDbF compared with similar FDCA-based polyesters.

Polyester	Elongation at Break (%)	Tensile Stress at Yield/MPa	Tensile Stress at Break/MPa	Young’s Modulus/MPa	Reference
PDDbF	732.38 ± 114.62	0.48 ± 0.06	0.97 ± 0.25	2.3 ± 0.22	this work
PDDF	130 ± 10	9.5 ± 1.1	10.8 ± 0.9	180.7± 16	[27]
PDeF	135 ± 17	10.6 ± 1.4	11.4 ± 1.2	201.9 ± 15	[27]

## Data Availability

The data presented in this study are available in this article and supplementary material.

## Supplementary Materials

The following supporting information can be downloaded at: https://www.mdpi.com/article/10.3390/molecules28104124/s1, Figure S1: FTIR spectrum of DEbF, Figure S2: ^1^H NMR spectrum of DEbF, Figure S3: ^13^C NMR spectrum of DEbF, Table S1: ^1^H NMR resonances [300 MHz, CDCl3-d] of PDDbF, Figure S4: ^13^C NMR spectrum of PDDbF, Table S2: ^13^C NMR chemical shifts assignments of PDDbF, Figure S5: DSC thermogram for PDDbF, Figure S6: XRD pattern of PDDbF, Figure S7: Loss modulus trace of PDDbF.

## Sample Availability

Samples of the compounds are available from the authors.
