# Supplementary material for: Highly Flexible Poly(1,12-dodecylene 5,5′-isopropylidene-bis(ethyl 2-furoate)): A Promising Biobased Polyester Derived from a Renewable Cost-Effective Bisfuranic Precursor and a Long-Chain Aliphatic Spacer
Source: Molecules. 2023 May 16;28(10):4124. doi: 10.3390/molecules28104124 (PMC10222078; doi:10.3390/molecules28104124)
Supplement: Supplementary file 1 [file molecules-28-04124-s001.zip › molecules-2349243-supplementary.pdf]

Highly Flexible Poly(1,12-dodecylene 5,5′-Isopropylidene-bis(ethyl 2-furoate)): A Promising Biobased Polyester Derived from a Renewable Cost-Effective Bisfuranic Precursor and a Long-Chain Aliphatic Spacer

Sami Zaidi [1,2], Abdelkader Bougarech [2], Majdi Abid [3], Souhir Abid [2,3], Armando J. D. Silvestre [1] and Andreia F. Sousa [1,4,*]

1 CICECO–Aveiro Institute of Materials and Department of Chemistry, University of Aveiro, 3810-193 Aveiro, Portugal
2 Faculty des Sciences, Laboratory de Chimie Appliquée HCGP, Université de Sfax, Sfax 3038, Tunisia
3 Chemistry Department, College of Science and Arts in Al-Qurayyat, Jouf University, Al-Qurayyat P.O. Box 756, Al Jouf, Saudi Arabia
4 CEMMPRE, Department of Chemical Engineering, University of Coimbra, 3030-790 Coimbra, Portugal
* Correspondence: andreiafs@ua.pt

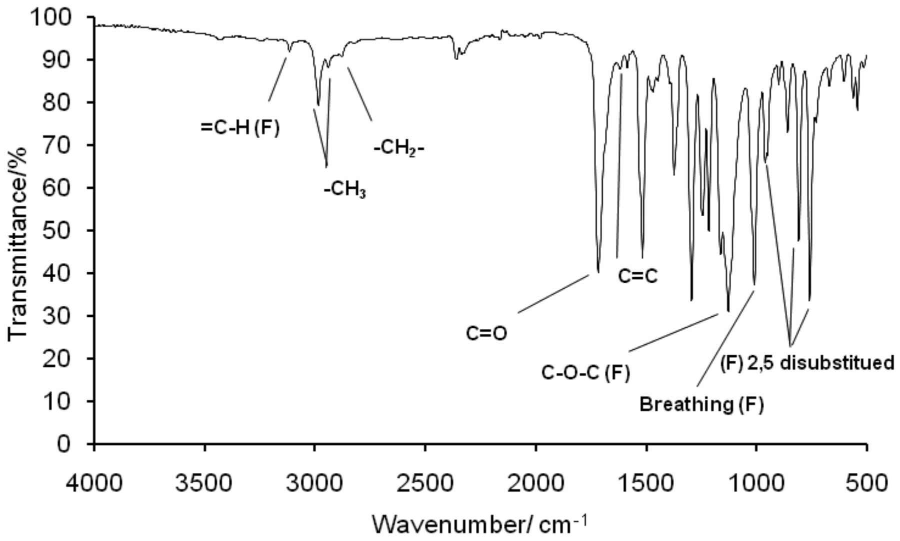

Figure S1. FTIR spectrum of DEbF.

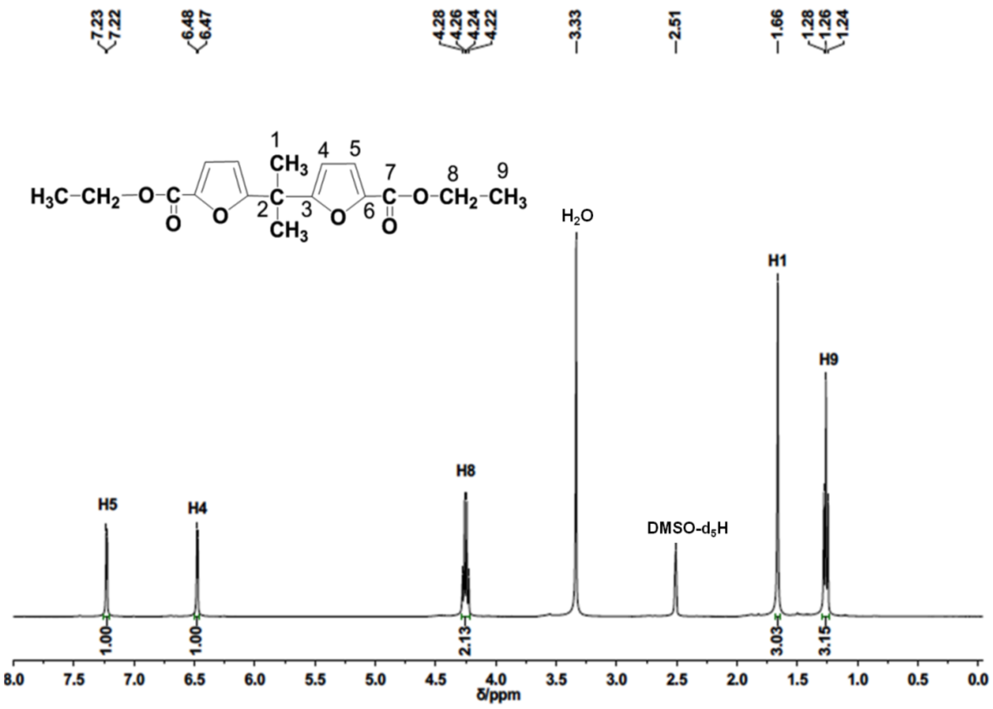

Figure S2. ^{1}H NMR spectrum of DEbF.

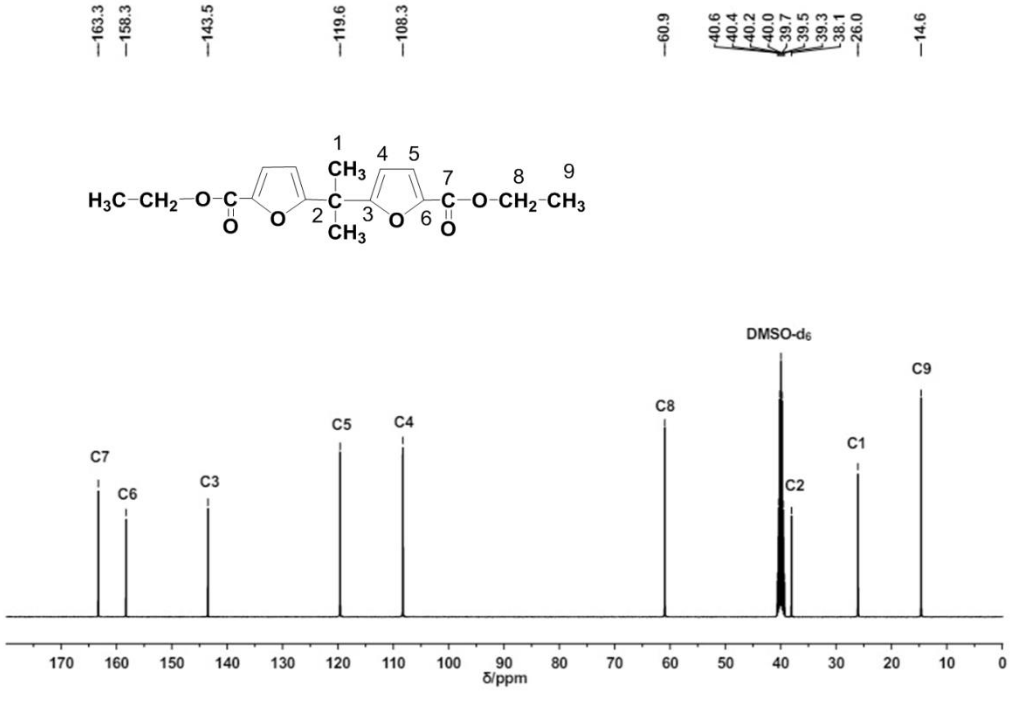

Figure S3. ^{13}C NMR spectrum of DEbF.

Table S1.¹H NMR resonances [300 MHz, CDCl3-d] of PDDbF.

Assignments	δ/ppm	multiplicity	Integration area
H5	6.97-6.98	d	1.00
H4	6.10-6.11	d	1.00
H8	4.15-4.20	t	2.19
H1,H9	1.59-1.66	m	5.85
H10,H11,H12,H13	1.20-1.30	m	9.91

d: doublet; t: triplet; m: multiplet.

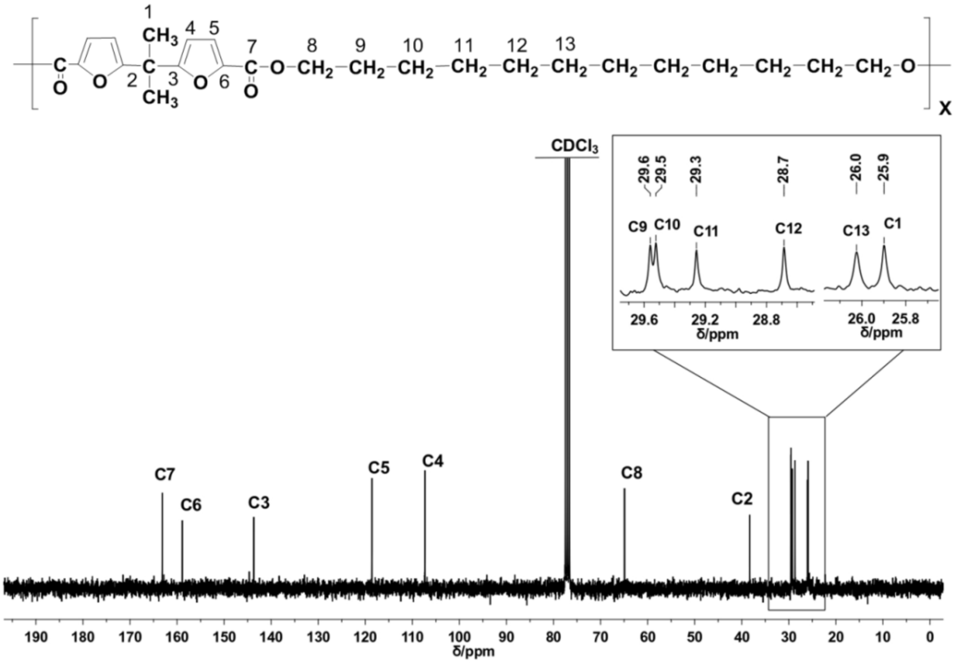

Figure S4. ¹³C NMR spectrum of PDDbF. The inset figure shows the different methylene and methyl proton resonances.

Table S2. ^{13}C NMR chemical shifts assignments of PDDbF.

Assignments	δ/ppm
C7	163.1
C6	158.9
C3	143.7
C5	118.6
C4	107.3
C8	64.9
C2	38.3
C9	29.6
C10	29.5
C11	29.3
C12	28.7
C13	26.0
C1	25.9

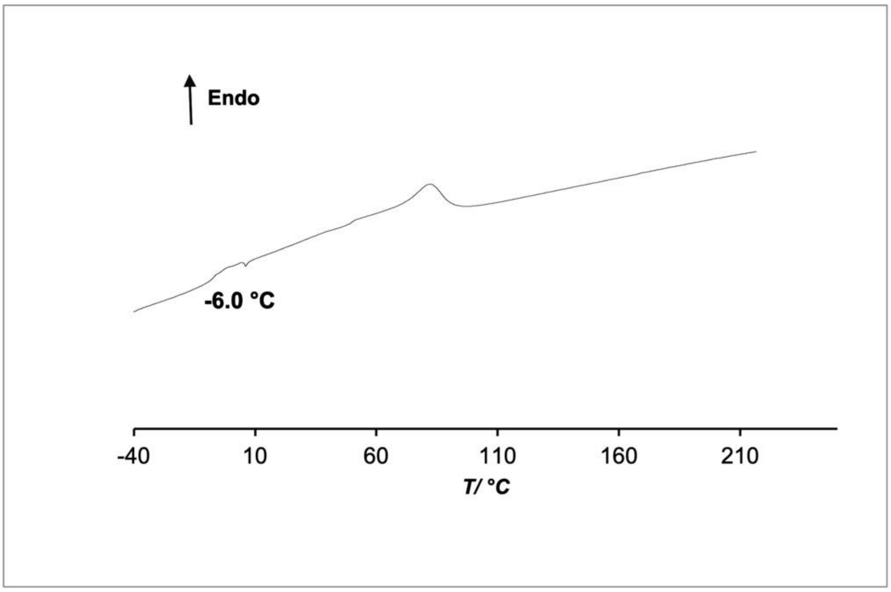

Figure S5. DSC thermogram for PDDbF.

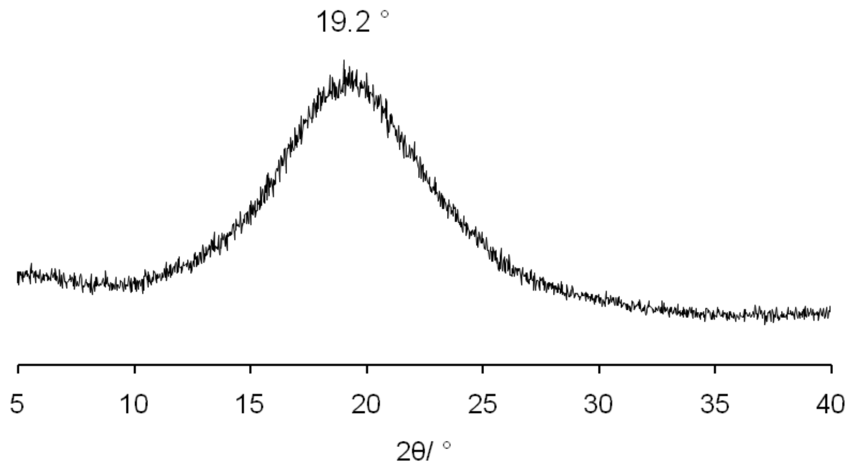

Figure S6. XRD pattern of PDDbF.

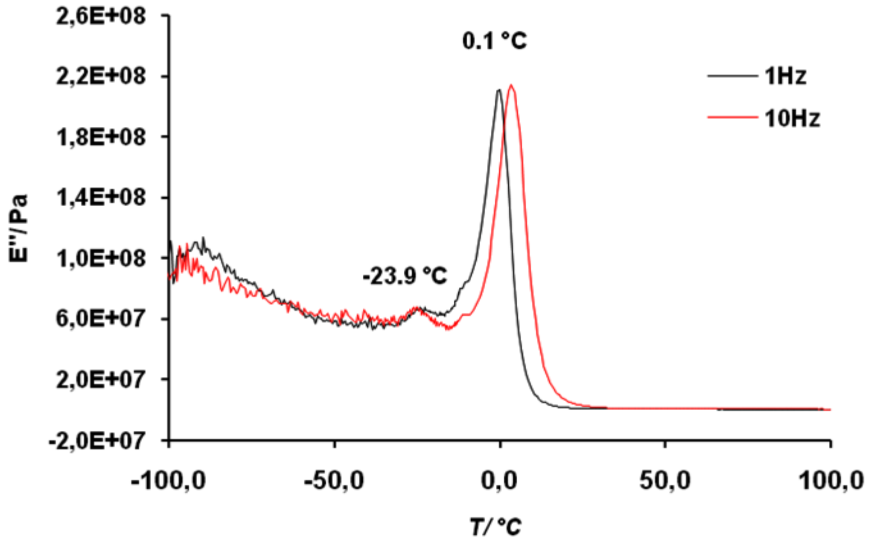

Figure S7. Loss modulus trace of PDDbF.
